# The impact of parasitism on resource allocation in a fungal host: the case of *Cryphonectria parasitica* and its mycovirus, Cryphonectria Hypovirus 1

**DOI:** 10.1002/ece3.3143

**Published:** 2017-06-23

**Authors:** Jérémie Brusini, Marta L. Wayne, Alain Franc, Cécile Robin

**Affiliations:** ^1^ Harbor Branch Oceanographic Institute Florida Atlantic University Fort Pierce FL USA; ^2^ BIOGECO INRA University of Bordeaux Cestas France; ^3^ Department of Biology University of Florida Gainesville FL USA

**Keywords:** evolution, host, life‐history traits, parasite, trade‐off, transmission, virulence

## Abstract

Parasites are known to profoundly affect resource allocation in their host. In order to investigate the effects of Cryphonectria Hypovirus 1 (CHV1) on the life‐history traits of its fungal host *Cryphonectria parasitica,* an infection matrix was completed with the cross‐infection of six fungal isolates by six different viruses. Mycelial growth, asexual sporulation, and spore size were measured in the 36 combinations, for which horizontal and vertical transmission of the viruses was also assessed. As expected by life‐history theory, a significant negative correlation was found between host somatic growth and asexual reproduction in virus‐free isolates. Interestingly this trade‐off was found to be positive in infected isolates, illustrating the profound changes in host resource allocation induced by CHV1 infection. A significant and positive relationship was also found in infected isolates between vertical transmission and somatic growth. This last relationship suggests that in this system, high levels of virulence could be detrimental to the vertical transmission of the parasite. Those results underscore the interest of studying host–parasite interaction within the life‐history theory framework, which might permit a more accurate understanding of the nature of the modifications triggered by parasite infection on host biology.

## 1 INTRODUCTION

Because of their ubiquity and their deleterious effect on host fitness (i.e., virulence), parasites are important drivers of the ecology and the evolution of their host populations (Dobson & Hudson, 1986; Price et al., 1986; Thompson, 2005), and even of entire ecosystems (Connell, 1978; Hudson, Dobson, & Lafferty, 2006; Janzen, 1970). The recent Zika virus outbreak in South America, and the emergence of invasive pathogens in agricultural systems such as the bacterium *Xylella fastidiosa* responsible for the olive quick decline syndrome in the Mediterranean basin, as well as the fungal pathogen *Guignardia citricarpa* in citrus cultures in South Florida, illustrate only a tiny sample of the threats to human activities caused by parasites. These case studies also underscore the need to study and understand the strategies that permit to host populations to evolve and to survive to parasite pressure.

Differential investment in life‐history traits is a powerful mechanism for organisms to adapt to ecological challenges. Life‐history theory stipulates that an organism's fitness is shaped by trade‐offs between traits such as growth, survival, and reproduction (Roff, 1992; Stearns, 1992). Optimal resource allocation among these traits should be favored by natural selection, given that an individual organism's resources are finite. Both empirical and theoretical studies have shown that parasite infection may alter host life‐history traits, causing an evolutionary response in host resource allocation (Agnew, Koella, & Michalakis, 2000; Chadwick & Little, 2005; Fredensborg & Poulin, 2006; Gandon, Agnew, & Michalakis, 2002; Hechinger, 2010; Heins & Baker, 2014). Fredensborg & Poulin (2006), investigated adaptation to a castrating parasite, the trematode *Maritrema novaezealandensis*, in the marine gastropod *Zeacumantus subcarinatus*. They found a significant negative correlation between host age at maturity and parasite prevalence, suggesting that hosts adapted to the parasite by shifting reproductive maturity earlier such that probability of reproduction prior to parasitism increases. In the cricket *Acheta domesticus*. Adamo (1999) observed an increase in egg laying following the activation of the host immune system by injection of inert components of the cell wall of the bacteria *Serratia marcescens*, again suggesting an acceleration of reproduction in the face of probable parasitism. Similar changes in life history in response to parasitism also exist in plants, as do many cases of variation in life‐history traits involved in resistance or tolerance have been reported in *Arabidopsis thaliana* (Kover & Schaal, 2002; Pagán, Alonso‐Blanco, & Garcia‐arenal, 2008; Salvaudon & Shykoff, 2013) as well as in other plant species (Bruns, Carson & May, 2012). Surprisingly, in fungi, no clear evidence of parasite‐driven modifications on fungal life‐history traits has yet been reported, although conversely host genetic identity was found to alter resource allocation balance in two fungal pathogens, the oomycete *Hyaloperonospora parasitica* (Heraudet, Salvaudon, & Shykoff, 2008) and the basidiomycete *Puccinia triticina* (Pariaud et al., 2013). Many fungi are pathogens of medical or agricultural importance; understanding how parasitism changes fungal life history could suggest new approaches to control of these destructive organisms via the use of biological agents.

Here, we propose to assess the existence of parasite‐driven modifications on fungal life‐history traits in the chestnut blight fungus, *Cryphonectria parasitica,* and its mycovirus (Cryphonectria Hypovirus 1 or CHV1), one of the best documented cases of parasitism of a fungal host. Previous work has documented the existence of significant genetic interactions between fungi and viruses from different lineages in this system (Peever, Liu, Cortesi, & Milgroom, 2000), as well as genotype‐by‐genotype‐by‐environment (G × G × E) interactions (Bryner & Rigling, 2011). However, the effect of CHV1 infection on fungal resource allocation has not been studied directly. In this study, we carried out a cross‐inoculation experiment consisting of 36 combinations of fungal and viral isolates. Growth, sporulation rate, and spore size of both infected and uninfected fungal isolates were measured, as well as vertical and horizontal transmission rates of the virus. A significant reversal of the correlation between *C. parasitica* reproduction and somatic growth was observed between virus‐free versus infected fungi. The relevance of these results on our understanding of *C. parasitica* /CHV1 interaction and the balance between life‐history traits in fungi are then discussed.

## 2 MATERIALS AND METHODS

### 2.1 Biological system

The biological system consists of three trophic levels: the plant host, the chestnut tree (*Castanea sp*.); the fungal parasite, *C. parasitica;* and the hyperparasite, the mycovirus CHV1. *C. parasitica* is an Ascomycete (Cryphonectriaceae family) and is the agent of the chestnut blight disease. This fungal pathogen caused the quasi‐extinction of the American chestnut (*Castanea dentata*) after its accidental introduction at the beginning of the last century to North America from Asia*. C. parasitica* was subsequently introduced to Europe a few decades after its introduction to North America (Dutech et al., 2012). However, in Europe, the effect of the fungal pathogen on the local populations of chestnut tree (*C. sativa*) was less dramatic because of the high prevalence of CHV1 (Milgroom & Cortesi, 2004). Infection by CHV1 reduces the deleterious effect of the fungus on its tree host, a phenomenon known as hypovirulence (Grente, 1981). Infection by CHV1 can be assessed by host phenotype: Virus‐infected mycelia are white and have slow, often deformed growth, whereas virus‐free mycelia are orange pigmented and have regular growth.

As is typical for Ascomycetes, *C. parasitica* reproduces both asexually via conidiospores and sexually through the production of ascospores. As ascospores do not transmit the virus (Anagnostakis, 1987; Carbone, Liu, Hillman, & Milgroom, 2004), we will not consider them further here. CHV1 is vertically transmitted solely by the asexual conidiospores, at a rate dependent on the genotypes of both fungus and virus (Bryner & Rigling, 2012; Peever et al., 2000). The virus is also horizontally transmitted between individuals after hyphal anastomosis (somatic fusion between two mycelia), which results in cytoplasmic mixing (Hickey, Jacobson, Read, & Glass, 2002). However, rates of horizontal transmission are reduced by the phenomenon of vegetative incompatibility: *In vitro,* transmission rates are high among individuals belonging to the same vegetative compatibility (vc) types, and low or null between individuals of different vc types (Liu & Milgroom, 1996).

### 2.2 Fungal virus‐infected isolates

Six isolates of *C. parasitica* infected by CHV1 (called hypovirulent isolates thereafter) and belonging to the same vc type, EU‐2 (Table 1), were used in this study. All six had the same allelic composition for ten microsatellite markers (Dutech, J.‐P, Fabreguettes, & Robin, 2008) and thus were assumed to belong to the same clonal lineage. Partial sequencing of the ORFA and ORFB in the CHV1 strains extracted from the six fungal isolates indicated that they belonged to four different genetic clusters as described by Feau, Dutech, Brusini, Rigling, & Robin (2014) (Table 1). Although two of the genetic clusters were represented by two different viruses each, sequencing revealed that viruses from the same cluster were genetically distinct from one another, as each contained unique SNPs.

**Table 1:** Geographical origin and viral clusters of the six hypovirulent isolates of *Cryphonectria parasitica* used in this study

Isolates	Geographical origin	CHV‐1 viral cluster (ORFA‐ORFB)	GenBank accession numbers (ORFA‐ORFB)
Doi12	Doissat, SW France	A3‐B3	JF790929‐JF795825
Gan20	Ganges, SE France	A3‐B3	JF790944‐JF795840
Doi1	Doissat, SW France	A4‐B3	JF790932‐JF795828
Doi21	Doissat, SW France	A1‐B1	JF790933‐JF795829
Gon37	Gonfaron, SE France	A2‐B1	JF790946‐JF795842
Doi88	Doissat, SW France	A1‐B1	JF790940‐JF795836

### 2.3 Cross‐inoculation of virus‐free isolates

The first step consisted in obtaining virus‐free fungal isolates from each of the six hypovirulent *C. parasitica* isolates. A concentrated suspension of conidiospores at 500 000 spore/ml in sterile water was collected from each isolate and 0.2 ml spread onto 15 ml of potato dextrose agar (PDA, Difco Laboratories, Detroit) in 9 cm Petri plates. After 48 hours of incubation at 25°C in the dark, the germinating spores were individually transferred to new Petri plates (10 spores per plate). Plates were then incubated for an additional two days at 25°C in the dark followed by two days under ambient conditions. Only colonies showing the characteristic orange pigmentation and regular growth of virus‐free isolates were retained. The same procedure was repeated three times over three generations of conidiospores. Only isolates showing no CHV1 infection symptoms for three consecutive generations were considered completely cured and were used as recipients for the cross‐inoculation experiment. Because conidiospores arise via asexual reproduction, each hypovirulent isolate and its derived cured isolates were genetically identical. Finally, each of the six cured isolates was infected by the six hypoviruses through hyphal anastomosis (i.e., somatic fusion between juxtaposed mycelia) using a previously described method (Liu & Milgroom, 1996). The six cured isolates were also included in the experiment, for a total of 42 fungal isolate (36 virus‐infected or hypovirulent isolates plus the six virus‐free fungal isolates).

### 2.4 Assessment of growth and reproduction traits

Three replicates of each the 42 fungal isolates were initiated on PDA agar with a plug of three‐day‐old cultures. After incubating for three days under standard conditions (22°C with a 18‐h/6‐h photoperiod ‐ fluorescent lamps at 150 micromols m^−2^ s^−1^), the radial length of each colony was measured. When colonies had a noncircular growth, the longest radius was measured. After a total incubation period of three weeks, conidiospores produced by each colony were collected by adding 25 ml of sterile water on the mycelium surface in each Petri plate. Petri plates were gently agitated for 30s, and one milliliter of the spore suspension was diluted with 24 ml of Isoton II (Coulter Counter^®^). The concentration of the diluted suspension was measured using a particle sizing and counting analyzer Multisizer^™^ 3 (Coulter Counter; 30 μm Aperture Tube), which provided the concentration in spores/ml (subsequently converted to numbers of spores), as well as the mean spore size for each spore suspension. Bacterial infection was found in two unrelated cultures, which were discarded and removed from further analysis.

### 2.5 Vertical and horizontal transmission of the viruses

The horizontal transmission of the viruses to a virus‐free recipient isolate was assessed for the 36 hypovirulent isolates. CHV1 transmission between mycelia of the same vc‐type is usually close to 100%. For this reason, the virus‐free recipient isolate was selected to belong to a different vc‐type than EU‐2. A reference isolate of the vc‐type EU‐8 was then chosen because of the relatively weak incompatibility reaction observed when paired with a mycelium of vc‐type EU‐2, thereby permitting CHV‐1 transmission at intermediate percentage frequencies (Cortesi, McCulloch, Song, Lin, & Milgroom, 2001) and thus maximizing our power to detect differences in transmission rates. Between 16 and 32 pairings were performed for each treatment combination. After two weeks of incubation at ambient conditions, the recipient isolates which exhibited the same phenotype as the infected donor isolate (i.e., no pigmentation and irregular growth) were paired with the original, virus‐free isolate to confirm infection as described in Brusini and Robin (2013).

Vertical transmission rates of the virus in the 36 hypovirulent isolates were also measured. For each fungal isolate, suspensions of conidiospores were collected as described above from three replicates grown in 4.5 cm PDA Petri plates for 1 day at 25C in the dark, followed by 2 days under ambient conditions. For each replicate, thirty germinating spores were transferred onto three 9‐cm Petri plates with PDA using a binocular microscope. After one‐week incubation under ambient conditions, the presence of virus in each single‐spore isolate was determined by assessing their phenotype (mycelium color and regularity of its growth). Rate was calculated as percentage of infected spores.

### 2.6 Data analysis

A principal component analysis followed by agglomerative hierarchical clustering with k‐means consolidation was performed on the average values per viral strain for the five measured traits in hypovirulent isolates (colony size at day 3, sporulation rate, spore size, horizontal transmission, and vertical transmission). Analyses were performed using the functions PCA and HCPC from the FactoMineR library in the R statistical software (Team R Development Core, 2009). Analysis of variance was performed (using SAS PROC GLM and type III sums of squares) to study the effect of the ORFB viral cluster, of the virus nested within ORFB cluster, of the host fungus and of their interactions (all factors were considered as fixed) on three life‐history traits: colony size at day three, sporulation rate, and spore size. Three replicates per fungus‐virus combinations were obtained for each of the three traits. Before performing these analyses, data were investigated for any violations of normality and homoscedasticity. Count data for number of spores produced after a three‐week incubation period (sporulation) did not conform to these assumptions; accordingly, these data were log transformed prior to further analysis. For virus vertical and horizontal transmission, only virus and fungus factors could be tested. Pearson's correlation tests were performed between CHV1 and *C. parasitica* fitness proxies as the Pearson correlation is robust for continuous non‐normal dataset without outliers (Chok, 2010). Bonferroni's correction was then used to address the problem of multiple comparisons. Finally, to directly compare the two correlation coefficients between infected and uninfected isolates, we used a Z‐test (Myers & Sirois, 2004).

## 3 RESULTS

### 3.1 Hierarchical cluster analysis of fungal impacted by the virus and viral traits

**Figure 1:**
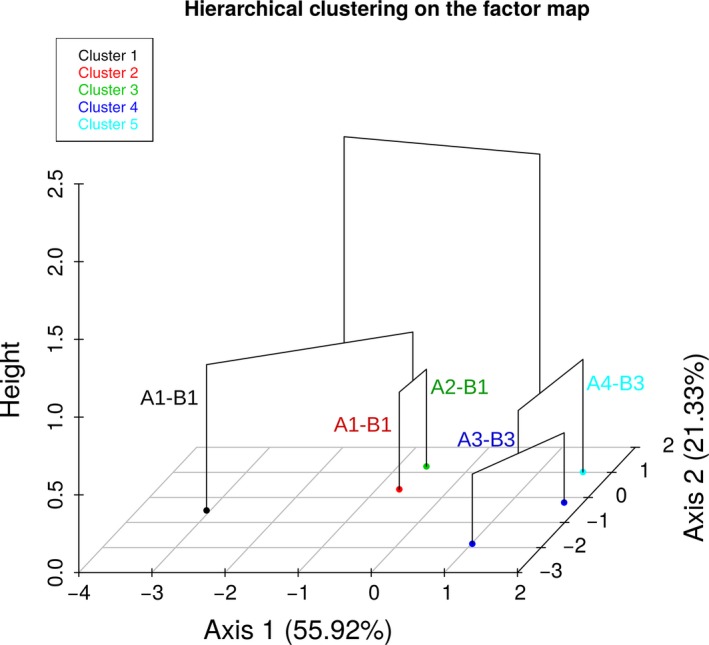
Representation of the hierarchical clustering on the average values of colony size at day 3, sporulation, spore size measurements, vertical transmission, and horizontal transmission for the six CHV1 isolates. On the factor map is represented the two first principal components (Axis 1 and Axis 2). Their respective contributions on the total variance explanation are into brackets. The height represents the inertia gain

The two first principal components (PCs) of the principal component analysis explained 77.25% of the total variance. Colony size at day 3 and sporulation rate contributed almost equally to PC1 (29.6% and 29.4% respectively). In contrast, PC2 was dominated by spore size (57.1%), followed by horizontal transmission (25.7%; see Table 2 for the complete list of contribution of each variable to PCs). Five clusters were identified, with the first partition splitting viruses according to their ORFB cluster (Figure 1).

**Table 2:** Contribution of each variable in the principal component analysis on the average values of the six CHV1 isolates. PC: principal components

Traits	PC1	PC2	PC3	PC4	PC5
Spore size	9.003	57.0961	0.0679	28.473	5.358
Colony size at day 3	29.651	7.951	0.0306	17.016	45.349
Sporulation	29.477	1.4612	12.714	15.517	40.829
Vertical transmission	15.60	7.776	74.245	0.510	1.862
Horizontal transmission	16.26	25.713	12.942	38.482	6.599

### 3.2 Viral transmission and impact of viral infection on host traits

Unsurprisingly given their different loading onto PC1 and PC2, horizontal and vertical transmission had fundamentally different genetic architectures. The vertical transmission of a virus was significantly dependent of the ORFB cluster to which the virus belongs, while horizontal transmission was not related to ORFB cluster (Table 3). Viruses belonging to the cluster B3 were vertically transmitted with a higher average rate (68%) than viruses of B1 cluster (54%).

**Table 3:** Analysis of variance of rates of horizontal and vertical transmission of CHV1 virus infecting different fungal isolates of *Cryphonectria parasitica*

Effect	*df*	Horizontal transmission		Vertical transmission	
		*F*	Pr > *F*	*F*	Pr > *F*
Virus	5	2.12	.102	9.00	<.0001
Fungus	5	1.38	.270	1.84	.142

Virus within the ORFB cluster had a significant effect on the colony size at day 3 for the fungal isolate with which it was associated (Table 4). For fungal growth, this effect was dependent on the ORFB cluster to which the virus belonged, such that viruses of cluster B1 decreased significantly more the growth of infected fungi than viruses of cluster B3. The fungus effect and its interaction with virus within the ORFB cluster were also significant. The sporulation rate of a virus‐infected fungus was significantly dependent on the virus and ORFB cluster factors, whereas the significant contributors to variation for spore size were the nature of the host fungus and its interaction with virus within the ORFB cluster.

**Table 4:** Analysis of variance of growth rate, asexual sporulation rate after log transformation, and spore size of hypovirulent isolates

Effect	*df*	Growth rate		Sporulation rate		Spore size	
		*F*	Pr > *F*	*F*	Pr > *F*	*F*	Pr > *F*
Virus (ORFB cluster)	4	5.22	.0009	3.22	.0172	1.08	.3706
ORFB cluster	1	248.45	<.0001	32.66	<.0001	2.58	.1124
Fungus	5	3.11	.0134	1.22	.3085	6.04	.0001
Fungus*virus(ORFB)	25	2.34	.0027	1.05	.425	2.00	.0123

**Figure 2:**
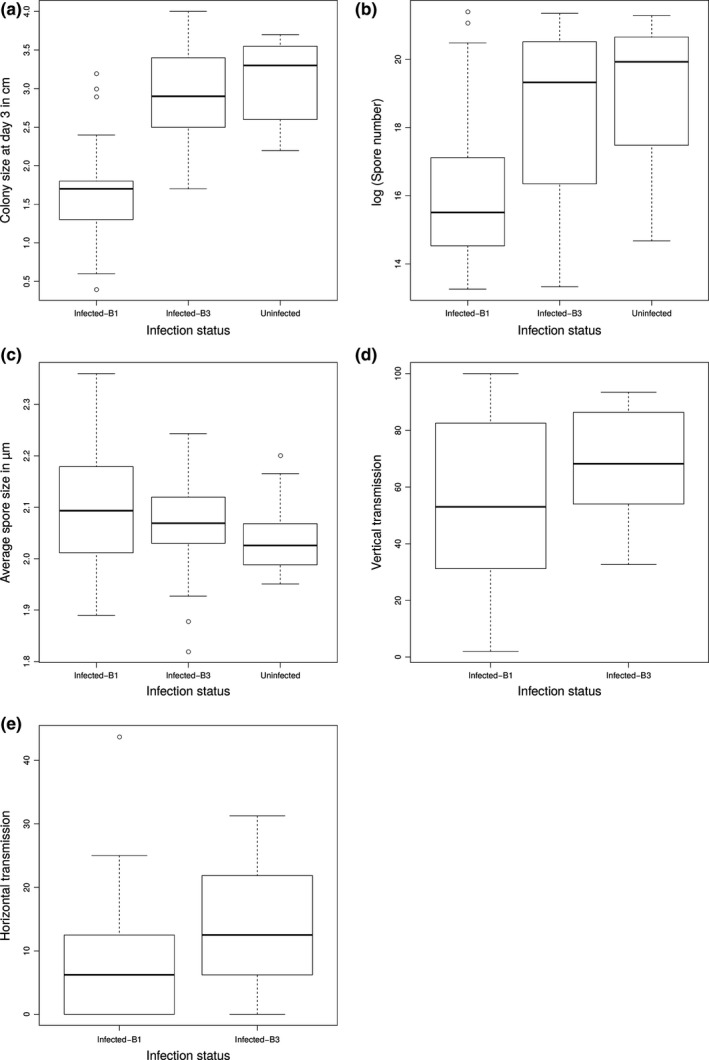
Boxplot of the distribution of fitness proxies values according to isolates infection status and the nature of the ORFB cluster of the CHV1 isolate

When infected by a virus of type B1, fungal isolates produced fewer spores (B1: 126 × 10^6^ spores/ml ± 49 × 10^6^ [SE], B3: 437 × 10^6^ spores/ml ± 69 × 10^6^ [SE]) and grew more slowly (B1: 1.64 cm ± 0.07 [SE], B3: 2.93 cm ± 0.07 [SE]) than when infected by a virus of type B3 (Figures 2a–c). Virus of type B3 also had higher vertical and horizontal transmission success than the B1 type (vertical transmission: B1: 53% ± 7 [SE], B3: 68% ± 4; horizontal transmission: B1: 53% ± 7 [SE], B3: 68% ± 4; Figure 2d,e).

### 3.3 Correlations between traits

**Figure 3:**
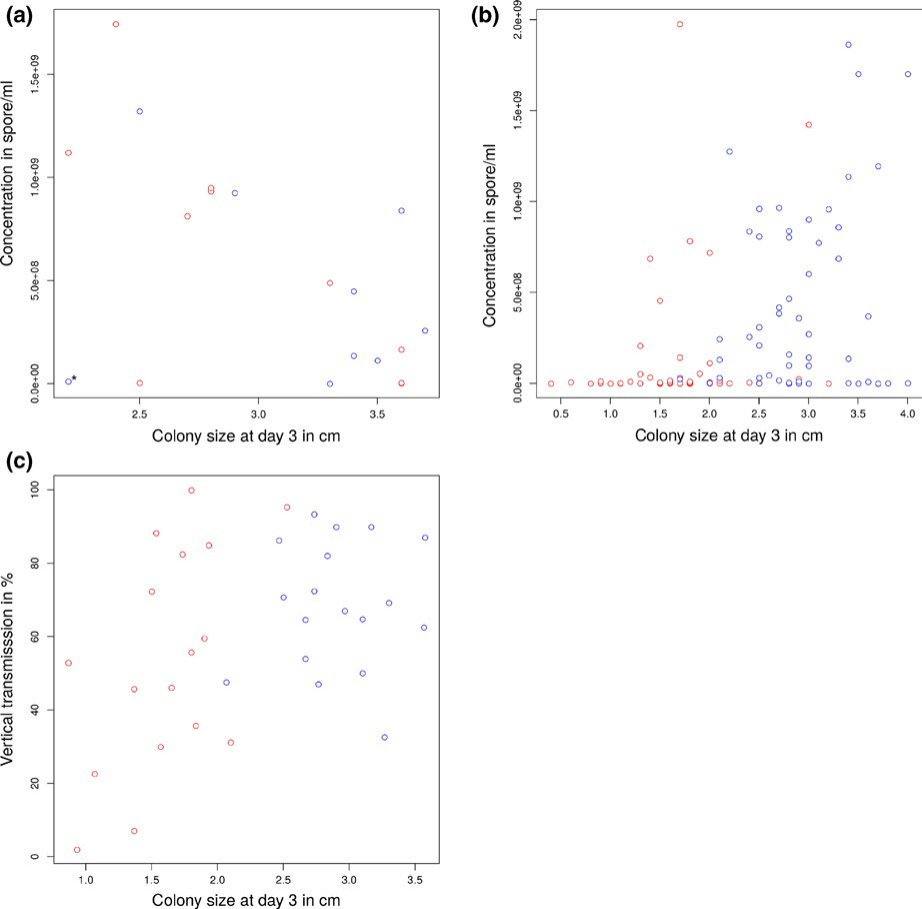
Scatter plots representing the significant correlation found in this study (a) between colony size at day 3 and concentration in spores in virus‐free recovered isolates, (b) between colony size at day 3 and concentration in spores in hypovirulent isolates and (c) between the average values of CHV1 vertical transmission and average colony size at day 3 in hypovirulent isolates. a: red and blue dots represent isolates that were previously infected by viruses of the B1 and B3, respectively; *: outlier. b and c: red and blue dots represent isolates infected by viruses of the B1 and B3, respectively

The infectious status of the fungal isolates affected the sign of the correlation coefficient between some fungal traits. For instance, a significant positive correlation was found between colony size and the number of spores produced by virus‐infected fungal isolates, whereas a significant negative correlation was observed in virus‐free isolates (Tables 5A and B; Figure 3a,b). A Z‐test indicated that the two correlation coefficients were indeed significantly different (*Z* = −3.47, *p *<* *.001). When calculated within each viral cluster, nonsignificant correlations between growth and spore load were found (B1: *r *=* *.21, *t* = 1.59, *df* = 52, *p *=* *.12; B3: *r *=* *.21, *t *= 1.52, *df* = 51, *p *=* *.13).

**Table 5:** Results of the correlation tests between CHV1 and *Cryphonectria parasitica* fitness proxies; A: in the 18 recovered virus‐free colonies; B: in the 106 hypovirulent colonies; C: when average traits values of each combination of CHV1/ *C. parasitica* isolates are calculated in order to include vertical and horizontal transmission measurements in the analysis. HT: horizontal transmission; VT: vertical transmission. Corr. *p*‐value: *p*‐values after Bonferroni's correction. Into brackets are indicated the correlation results without outliers (see Figure 3)

Variable 1	Variable 2	*t*	*N*	*r*	*p*‐value	Corr. *p*‐value
A						
Colony size	Sporulation	−2.57 (−3.92)	17 (16)	−.53 (−.70)	.02 (.001)	.06 (.003)
Colony size	Spore size	−0.5 (0.16)	17 (16)	−.12 (.04)	.62 (0.87)	1 (1)
Spore size	Sporulation	−1.65 (−1.3)	17 (16)	−.37 (−.31)	.12 (0.21)	.36 (.63)
B						
Colony size	Sporulation	4.26	105	.38	<.001	<.001
Colony size	Spore size	−1.40	105	−.14	.16	.48
Spore size	Sporulation	−0.91	105	−.09	.36	1
C						
HT	VT	0.12	30	.02	.9	1
HT	Colony size	1.02	31	.18	.31	.93
HT	Sporulation	1.12	31	.20	.27	.81
HT	Spore size	−1.91	31	−.32	.07	.21
VT	Colony size	2.74	33	.43	.01	.03
VT	Sporulation	1.00	33	.17	.32	.96
VT	Spore size	0.30	33	.05	.77	1

A significant and positive correlation was observed between the vertical transmission of virus and colony size of infected fungi (Table 5C, Figure 3c). While this correlation was also significant and positive when considering B1 viruses only (*r *=* *.54; *t *= 2.46, *df* = 15, *p *=* *.03), no significant relationship was found for the B3 viruses (*r *=* *.06, *t *= 0.25, *df* = 16, *p *=* *.81).

## 4 DISCUSSION

Resource allocation between fecundity and growth represents a classic life‐history trade‐off (Roff, 1992; Stearns, 1992). A key assumption is that an increase in resource investment devoted to current reproduction will reduce the amount of resources available for future reproduction or somatic growth. Such trade‐offs have been reported in animals (Creighton, Heflin, & Belk, 2009; Kubička & Kratochvíl, 2009; Ohbayashi‐Hodoki & Shimada, 2005; Reznick, 1983; Yund, Marcum, & Stewart‐Savage, 1997) and in plants (Houle, 1999; Obeso, 2002; Silvertown & Dodd, 1999; Sletvold & Ågren, 2015). However, investigations of resource allocation in fungi appear to be scarce (Bruns et al., 2012) and are often oriented toward the study of trade‐off between a fungal pathogen and its host (Heraudet et al., 2008; Kover, 2000; Pariaud et al., 2013). Our data demonstrating a negative correlation in virus‐free *C. parasitica* isolates between asexual sporulation and mycelial growth extends support for the hypothesis of trade‐offs between current reproduction and somatic growth to a new kingdom of multicellular organisms. Interestingly, the opposite result was found for hypovirulent isolates, where the correlation analysis showed a positive association between asexual reproduction and mycelial growth when *C. parasitica* was infected by CH1.

Two different hypotheses can be invoked to identify the mechanism behind a correlation shift between virus‐free and virus‐infected individuals. Host adaptive plasticity in resource allocation between traits may reduce the negative effect of parasite infection on host fitness by increasing its tolerance to infection. As a result, parasitized hosts need to allocate more resources to reproductive functions to counterbalance parasite detrimental effects, especially by accelerating the onset of reproduction so as to outpace the detriment of somatic growth (Agnew et al., 2000; Forbes, 1993). However, parasite manipulation may also explain modifications in resource allocation in response to parasitism (Fredensborg & Poulin, 2006). For both interpretations—host tolerance or parasite manipulation—an increase in reproductive function is expected to be energetically costly and to impact negatively on other host fitness proxies. Surprisingly, in virus‐infected isolates, the only significant correlation involving asexual sporulation rate was a positive correlation with mycelial growth. Asexual sporulation is the dominant mode of transmission of CHV1 via vertical transmission, and so is critical to CHV1 fitness. Any shift in the balance of resources that increases *C. parasitica* asexual sporulation rate would then benefit CHV1. For instance, the sexual castration that has been reported in some hypovirulent *C. parasitica* isolates (Zhang, Baasiri, & Van Alfen, 1998) could be interpreted as redirection of resources away from traits not benefiting viral fitness. The observation of a positive relationship between somatic growth and asexual reproduction in hypovirulent isolates could then suggest that CHV1 when infecting *C. parasitica* releases in its host a constraint between two important life‐history traits—that we found to be negatively correlated in uninfected isolates—in order to benefit to its own fitness. However, it has already been demonstrated that at the population level two traits in competition can show a positive correlation when the population is scattered over environments of different qualities (van Noordwijk & de Jong, 1986). In the present case—in vitro culture in controlled environment— *C. parasitica* environment quality is entirely defined by the nature of the viral lineage which consisted either in the highly virulent viruses from the cluster B1 or in the mildly virulent viruses from the cluster B3. A way to investigate whether this positive relationship is a biological reality or an experimental artifact would involve cultivating hypovirulent *C. parasitica* isolates on chestnut stems from different genetic background. Brusini and Robin (2013) have already demonstrated that CHV1 horizontal transmission was drastically modified when *C. parasitica* is cultivated on its natural host rather than on an artificial substrate, illustrating the need to assess chestnut tree impact on other important CHV1 and *C. parasitica* life‐history traits such as vertical transmission and somatic growth.

By assessing the genetic diversity of CHV1 populations in France and Spain, Feau et al. (2014) provided an improved framework for the classification of CHV1 strains based on the comparison of ORFA and ORFB partial sequences. In the present work, phenotypic assays were carried on CHV1 isolates representing four of five of the main ORFA clusters and two of three of the main ORFB clusters revealed by Feau et al. (2014). Results from principal component analyses strongly suggested that most of the phenotype traits variance between CHV1 strains was explained by the nature of the ORFB. Chen, Geletka, and Nuss (2000) already pointed out the major role of the nature of the ORFB for the difference in the CHV1‐mediated alterations of fungal colony sporulation and growth using chimeric viruses generated from two viral strains. Based on the sample of viruses used in this present study, our results suggest that the classification of CHV1 strains according to the host growth and asexual sporulation could represent a noncostly and efficient way to identify CHV1 strains belonging to different ORFB clusters.

Our study confirms the existence of strong differences in the intensity of the deleterious effects on host fitness (i.e., virulence) caused by CHV1 infection from different lineages (Bryner & Rigling, 2012; Chen & Nuss, 1999; Peever et al., 2000; Robin, Lanz, Soutrenon, & Rigling, 2010). It also provides evidence that average spore size in hypovirulent isolates is another trait affected by CHV1 lineage. It would be interesting to investigate whether larger spore sizes constitute a selective advantage, such as an increase in germination success of infected spores, or if this observation just consists in a neutral byproduct of the alteration in somatic growth observed in fungi infected by B1 cluster viruses. This information would shed some light on the mechanism driving virulence evolution in CHV1, which is a rare example of virulent mycovirus. Some authors suggested that CHV1 virulence could be the direct consequence of the low vc‐type diversity of European population of *C. parasitica* (Robin & Heiniger, 2001), allowing virulence to be associated with a better horizontal transmission as predicted by the trade‐off hypothesis for the evolution of parasite virulence (Brusini, Robin, & Franc, 2011; Milgroom, 1999). This classical theory states that virulence is the unavoidable consequence of the within host parasite reproduction that is needed to assure its future transmission (Anderson & May, 1982). However, Michalakis, Olivieri, Renaud, and Raymond (1992) also hypothesized that CHV1 virulence is positively selected because the phenomenon of hypovirulence constrains *C. parasitica* to reach an evolutionary stable level of virulence, allowing the chestnut tree to survive fungal infection. Interestingly, Bryner and Rigling (2012) identified a positive correlation between the horizontal transmission of tree CHV1 isolates and their effects on *C. parasitica* growth. Because each isolate was belonging to a different viral lineage, the authors suggested that a mechanism similar to the trade‐off hypothesis was responsible for the difference in virulence observed between CHV1 lineages. Here, the comparison of the viral lineages effects based on the fitness proxies of 36 virus/host associations showed that horizontal transmission was not significantly correlated with any of the virulence proxies tested. Moreover, contrary to the results of Bryner and Rigling (2012) where the most virulent CHV1 strain presented the highest horizontal transmission rate, we found that most virulent CHV1 isolates (B1 cluster) on average had a lower horizontal transmission than the mildly virulent CHV1 isolates (B3 cluster). The only significant correlation involving transmission found in the 36 virus/host associations tested here was a positive correlation between vertical transmission and host colony size. This result is consistent with a tight association between the fitness of the host and the fitness of its vertically transmitted parasite, suggesting that vertical transmission drives down parasite virulence as expected by the literature (Day & Proulx, 2004; Ewald, 1987; Lipsitch, Siller, & Nowak, 1996). Several experiments in different host–parasite systems have confirmed that vertically transmission usually selects for less virulent parasites (Bull, Molineux, & Rice, 1991; Magalon, Nidelet, Martin, & Kaltz, 2010; Pagán, Montes, Milgroom, & García‐Arenal, 2014; Stewart, Logsdon, & Kelley, 2005). This relationship is even suspected to be responsible for the general avirulence of mycoviruses, especially when high vc‐type diversity in host populations limits the occurrence of horizontal transmission (Brusini et al., 2011). A similar and significant relationship was also found when restricting the analysis to CHV1 isolates belonging to the cluster B1. The fact that no significant correlation was observed in viruses from the cluster B3 could be seen as a lack of power due to a small sample size. However, it also raises the question of whether the evolution of virulence in those two CHV1 clusters is driven by two different forces. Similar investigations with a higher number of virus isolates would be needed to distinguish between these two hypotheses.

This study illustrates how fungi and notably ascomycetes can be good study models to investigate the nature of trade‐offs between life‐history traits. Their ease of culture (culture on gel media is possible for many species), their fast growth rate, and the ability to generate clonal replicates via asexual spores or vegetative cuttings represent several assets to the experimental assessment of environmental factors effects on resource allocation in multicellular organisms. It also highlights the necessity of including the third protagonist of the tritrophic interaction—the chestnut tree—in further studies in order to provide a better understanding on the mechanisms responsible for the evolution of European CHV1 lineages toward different level of virulence.

## CONFLICT OF INTERESTS

None declared.
